# Association Between Serum Vitamin D Levels and Parkinson's Disease: A Systematic Review and Meta-Analysis

**DOI:** 10.3389/fneur.2018.00909

**Published:** 2018-11-12

**Authors:** Xiaoyue Luo, Ruwei Ou, Rajib Dutta, Yuan Tian, Hai Xiong, Huifang Shang

**Affiliations:** ^1^Department of Neurology, West China Hospital, Sichuan University, Chengdu, China; ^2^Department of Geriatrics, The Fourth Affiliated Hospital of Sichuan University, Chengdu, China

**Keywords:** Parkinson's disease, vitamin D, meta-analysis, observational studies, motor symptom

## Abstract

**Background:** Vitamin D is an important secosteroid which is involved the development and regulation of brain activity. Several studies have focused on exploring the relationship between serum vitamin D levels and Parkinson's disease (PD), but the conclusion remains ambiguous.

**Methods:** We searched observational studies that explored the association between serum vitamin D levels and PD based on PubMed, EMBASE and Cochrane library from inception through to January 2018. The quality of included studies was evaluated by using Newcastle-Ottawa Scale (NOS). Statistical analysis of this meta-analysis was performed by Stata version 12.0 and R software.

**Results:** Twenty studies with a total of 2,866 PD patients and 2,734 controls were included. Compared with controls, PD patients had lower serum vitamin D levels (WMD −3.96, 95%CI −5.00, −2.92), especially in higher latitude regions (WMD −4.20, 95%CI −5.66, −2.75). Assay methods contributed significantly to high heterogeneity. Furthermore, PD patients with deficient vitamin D levels had advanced risk (OR 2.08, 95%CI 1.35, 3.19) than those patients with insufficient ones (OR = 1.73, 95%CI 1.48, 2.03). In addition, serum vitamin D levels were also related to the severity of PD (WMD −5.27, 95%CI −8.14, −2.39) and the summary correlation coefficient showed strongly negative correlation (*r* = −0.55, 95%CI −0.73, −0.29). Moreover, the pooled correlation coefficient revealed that serum vitamin D levels were also negatively correlated to the Unified Parkinson's Disease Rating Scale III (UPDRS III) (*r* = −0.36, 95%CI −0.53, −0.16), but did not correlate with the duration of PD (*P* = 0.37) and age of patients (*P* = 0.49).

**Conclusion:** Serum vitamin D levels are inversely associated with the risk and severity of PD. Our results provided an updated evidence of association between low vitamin D levels and PD and prompt the adjunctive therapeutic decisions about vitamin D replacement in PD.

## Introduction

Parkinson's disease (PD) is a common complex neurodegenerative disorder characterized by a variety of motor and non-motor symptoms. As the disease progresses, PD symptoms can significantly affect the quality of life. Recently, it was reported that the median age-standardized annual incident rate of PD has raised to 14 per 100,000 people in high-income countries (1), indicating that the burden of developing PD within a family is on the rise. To date, the exact pathogenesis of PD is still unclear, and no curative treatment is available. Thus, slowing down the progression of PD is of utmost importance.

Vitamin D is a group of steroid derivatives, which can regulate the metabolism of calcium and phosphate. Recently, there are increasing evidences which show that Vitamin D plays an important role in cell modulation, such as proliferation, differentiation, immunoregulation and so on (2). In addition, an animal study found that vitamin D might have a neuroprotective function (3). Vitamin D deficiency is common in PD (4). However, it is argued whether vitamin D insufficiency is related to the etiology of PD. Two recent longitudinal studies suggested that the reduced serum vitamin D levels are significantly associated with risk of developing PD (5, 6). This evidence provides the possibility of evaluating the risk of PD by using serum vitamin D levels as a biomarker.

Although three previous meta-analysis have focused on the relationship between serum vitamin D levels and PD (7–9), it did not include enough studies, extract all related data, and analyze the association between possible confounding factor and the indicator of PD progression. In our study, we therefore enlarged the number of studies and conducted a deeper systematic review and meta-analysis to assess the association between the serum vitamin D levels and PD, not only the susceptibility but also the severity of PD.

## Methods

### Search strategy

We searched the major biomedical databases, including PubMed, EMBASE, and Cochrane library for all related studies published before January 2018. The keywords used for searching were “Parkinson's disease” or “PD” combined with “vitamin D,” “vit D,” “cholecalciferol,” “ergocalciferols” “hydroxycholecalciferol” or “ergocalciferol.” To avoid missing literatures, we also looked through the references of relevant articles. Two researchers (Xiaoyue Luo and Yuan Tian) filtered the articles independently to identify qualified articles. If any disagreement or conflict, the third researcher (Ruwei Ou) decided whether the article was included in this meta-analysis. Our meta-analysis was registered with PROSPERO (registration number CRD42018086043).

### Definitions

All recruited patients met the diagnosis criteria of PD, we used the Unified Parkinson's Disease Rating Scale III(UPDRS III) to assess the motor symptoms. The severity of PD was defined on the basis of the Hoehn & Yahr (H&Y) scale and scores >3 indicated severe stages of PD (10). The controls were age-matched healthy individuals. Serum 25-hydroxyvitamin D was taken as a indicator of vitamin D status worldwide (11). Serum 25(OH)D concentration <20 ng/ml (50 nmol/l) was regarded as vitamin D deficiency, between 20 ng/ml (50 nmol/l) and 30 ng/ml (75 nmol/l) was considered as vitamin D insufficiency, and above 30 ng/ml (75 nmol/l) was considered as the normal level (11). The unit of nmol/l was converted to the value of ng/ml in our meta-analysis.

### Selection and exclusion criteria

**Figure 1:**
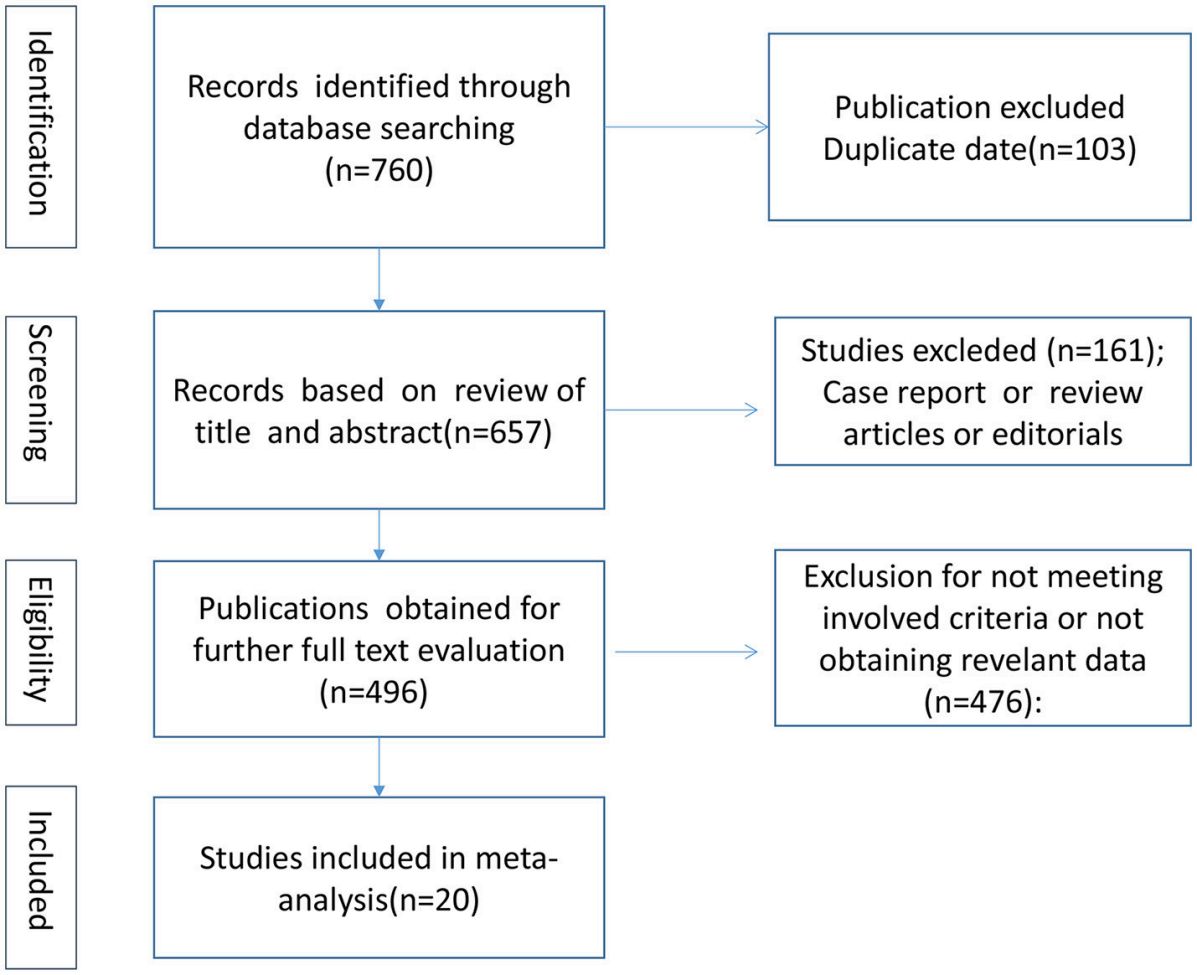
Flow diagram of search process and identified studies.

We finally included 20 articles showed in Table S1 in Supplementary Material by scanning the titles, abstracts and further reviewing the full texts. The flow chart of selection process is presented in Figure 1. All of the included articles met the following criteria: (1) cohort, case-control, or cross-sectional study; (2) to investigate the association between serum 25(OH)D levels and PD; (3) higher quality papers; and (4) published in English. The exclusion criteria were listed as follows: (1) case reports, reviews or editorial; (2) cells or animal studies; (3) the serum levels of vitamin D calculated by other indicators and not by 25(OH)D; and (4) incomplete data.

### Data extraction

The following data was extracted including first author, publication year, study design, sample size, methods of test, latitude, H&Y stages, UPDRS III scores, serum vitamin D levels and so on. To reduce mistakes and bias, two authors extracted data separately and identified by a third researcher. If the articles did not provide enough data of our interest, we tried our best to contact the author.

### Quality evaluation

We used Newcastle-Ottawa Scale (NOS) (12) to assess the quality of evidence. It contains three parts which includes selection (0–4 stars), comparability (0–2 stars) and exposure/outcome (0–3 stars). The NOS scores of 1–3, 4–6, and 7–9 indicated low, medium and high quality of studies, respectively (13). As displayed in Table S2 in Supplementary Material, this meta-analysis consisted of articles with stars ≥6 and could be considered as higher methodological quality. Any dissension among researchers was resolved by final discussion to reach a consensus.

### Statistical analyses

For dichotomous data, the odds ratio (OR) and 95% CI were pooled. For continuous data, the mean difference (MD) or the standardized mean difference (SMD) and 95% CI were calculated. In addition, we also combined correlation coefficient (r) using inverse variance method as reported before (14). Heterogeneity was evaluated by Chi^2^ test and *I*^2^ squared. *I*^2^ > 50% or *P* < 0.1 represented substantial heterogeneity and random effects model was choosen under this circumstance. Otherwise, fixed effects model was utilized. If high heterogeneity existed, subgroup and meta regression analysis was carried out. Sensitivity analysis was also done by removing the study one by one, switching effects model and exchanging of statistical values. The publication bias was estimated by Egger's test and funnel's plot if included papers ≥10 according to Cochrane handbook. All statistical analysis of this meta-analysis was performed by Stata version 12.0 (StataCorp LP, College Station, TX, USA) and R software (https://www.r-project.org/).

## Results

### Search results and study characteristics

A total of 760 articles were selected according to the search strategy mentioned before (Figure 1). After we reviewed the titles, abstracts and full texts, 20 studies (15–34) including 2,866 PD patients and 2,734 controls met our inclusion criteria. Fifteen articles (15, 17–19, 21, 23–26, 28, 29, 31–34) provided serum 25(OH)D levels of PD patients compared with those of controls. Seven included articles (17, 18, 21, 29, 31–33) analyzed vitamin D insufficiency between PD patients and controls, while 10 articles (17–21, 28, 29, 31, 33, 34) assessed vitamin D deficiency in PD patients compared with controls. Six included papers (16, 17, 19, 25, 26, 30) evaluated the association between serum vitamin D levels and the severity of PD. At last, 6 essays (19, 22, 25–28) were identified for exploring the correlative factors of vitamin D, including H&Y stages, UPDRS III scores, age of patients and disease duration.

### Serum vitamin D levels in PD patients

**Figure 2:**
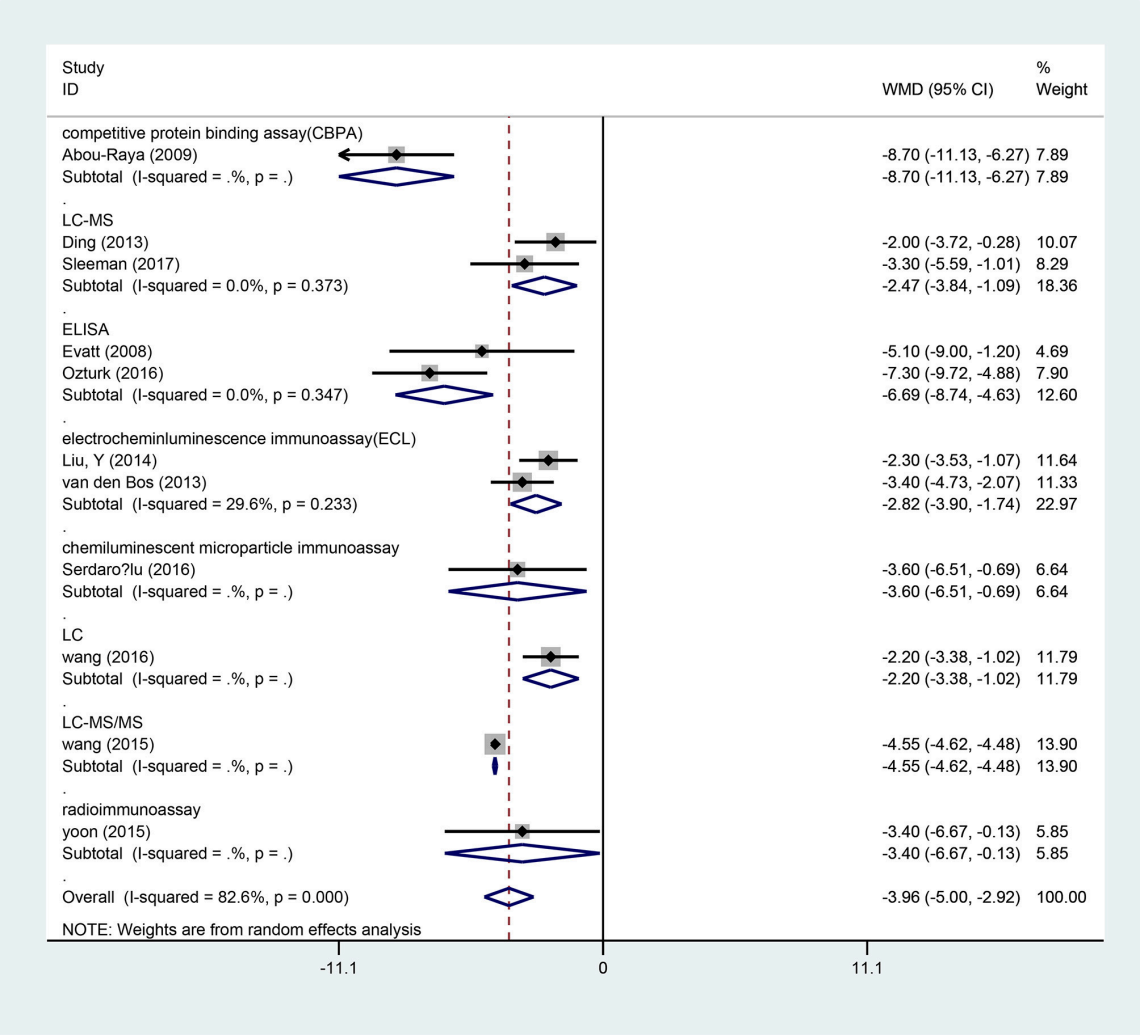
Forest plot of vitamin D levels between PD patients and controls divided by assay methods.

**Figure 3:**
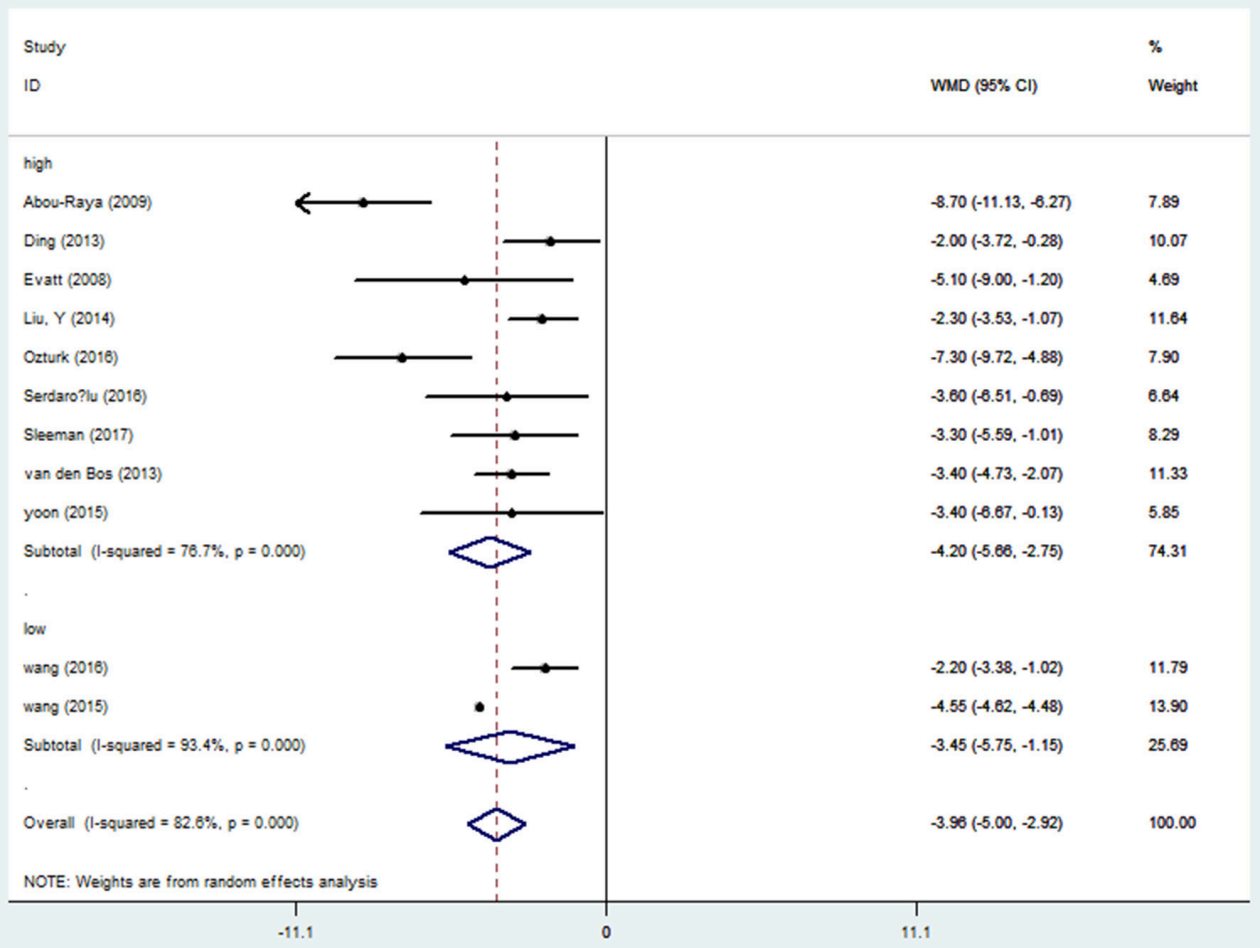
Forest plot of vitamin D levels between PD patients and controls divided by latitude.

Fifteen studies (15, 17–19, 21, 23–26, 28, 29, 31–34) provided data of serum vitamin D levels between PD patients (*n* = 2,436) and age matched controls (*n* = 2,567). We only included the paper with the most complete description of data (23) since four papers had the same first author's name (Sato Y) (23–26). Our results showed that the serum vitamin D levels in PD patients were significantly lower than those in controls (WMD −5.64, 95%CI −8.65, −2.62), which had marked heterogeneity (*I*^2^ = 98.6%). Further meta funnel research found the funnel plot was asymmetric and the study of Sato et al. (23) deviated much from it. After we removed this study, however, the result did not change (WMD −3.96, 95%CI −5.00, −2.92, *I*^2^ = 82.6%, Figure 2). To explore the high heterogeneity, the subgroup analysis of assay methods was conducted. It could extremely explain the source of heterogeneity. We also studied the subgroup analysis of different latitude, which indicated that PD patients in higher latitude area (WMD −4.20, 95%CI −5.66, −2.75) had lower serum vitamin D concentrations than those in lower latitude ones (WMD −3.45, 95%CI −5.75, −1.15, Figure 3). Although high heterogeneity existed, the meta regression showed that the latitude degree was not the main source of heterogeneity (adjust-R^2^ = 16.26%, *P* = 0.636). Also, publication bias was detected by Egger's test and the outcome showed no significant bias (*P* = 0.30). Sensitivity analyses revealed the pooled effects were robust, and so did the trim and fill method.

### Vitamin D insufficiency and PD risk

**Figure 4:**
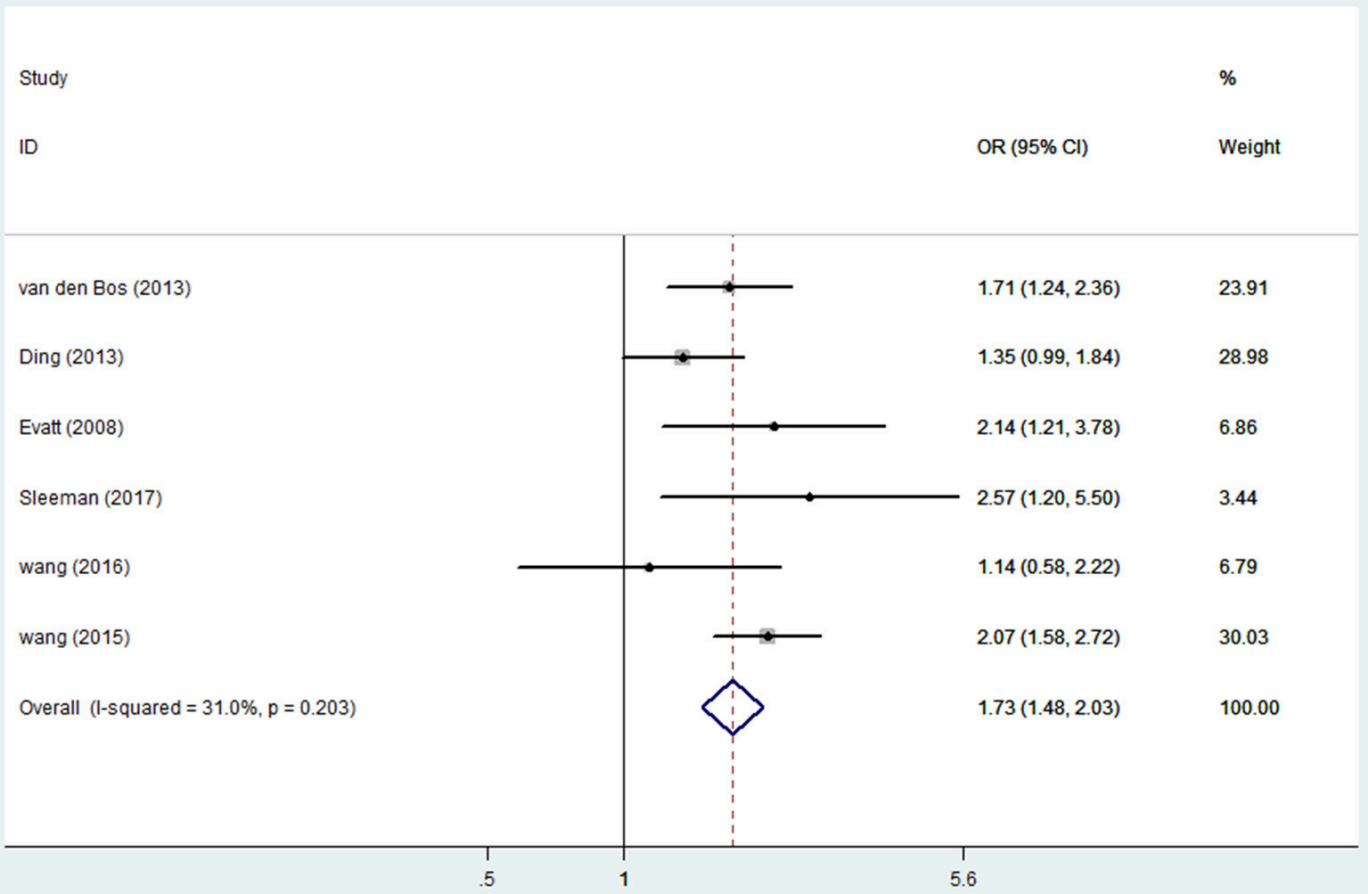
Forest plot of insufficient vitamin D levels and risk of PD.

Seven articles (17, 18, 21, 29, 31–33) including 1,613 PD patients and 2,026 controls evaluated the influence of vitamin D insufficiency on the risk of PD. Due to statistical heterogeneity (*I*^2^ = 70.9%), the random effects model was selected. The results revealed a pooled OR of 1.52 (95%CI 1.10, 2.10, *P* = 0.012), suggesting that vitamin D insufficiency can increase the risk of PD. Due to the limited number of articles, publication bias was not computed. The sensitivity analyses showed no excessive study could alert the final consequence. However, when we removed the study of Ozturk et al. (21), the heterogeneity (*I*^2^) decreased to 31% (OR = 1.73, 95%CI 1.48, 2.03, Figure 4).

### Vitamin D deficiency and PD risk

**Figure 5:**
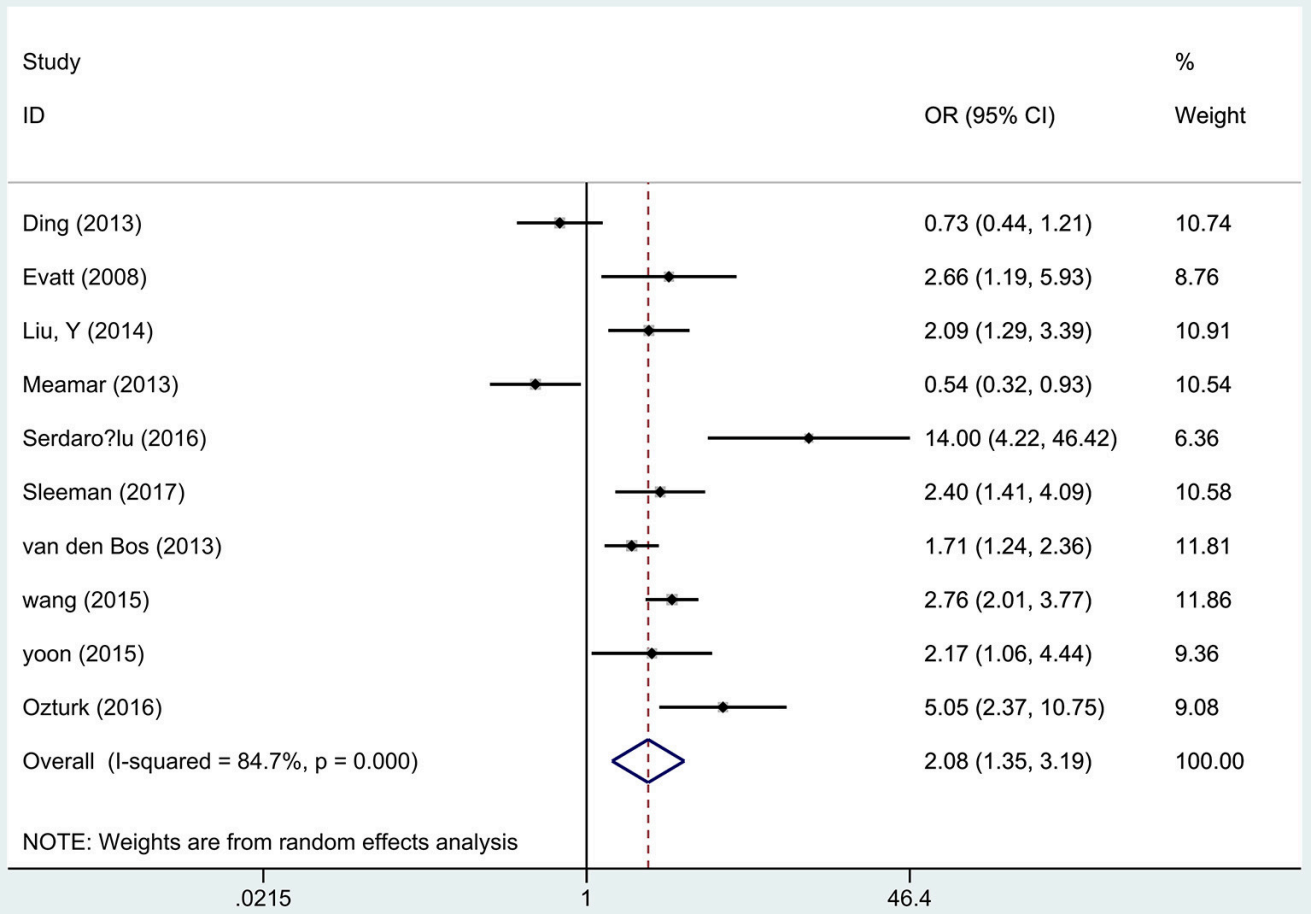
Forest plot of deficient vitamin D levels and risk of PD.

Ten articles (17–21, 28, 29, 31, 33, 34) including 1,879 PD patients and 1,978 controls reported the association between the vitamin D deficiency and the risk of PD. A remarkable association was found that vitamin D deficient subjects had a 2-fold increased risk of PD than controls using the random effects analysis (OR 2.08, 95%CI 1.35, 3.19, *I*^2^ = 84.7%, Figure 5), which was also higher than insufficient groups. In addition, Egger's test identified no publication bias (*P* = 0.572). Sensitivity analyses comfirmed the similar results.

### Serum vitamin D levels and the severity of PD

**Figure 6:**
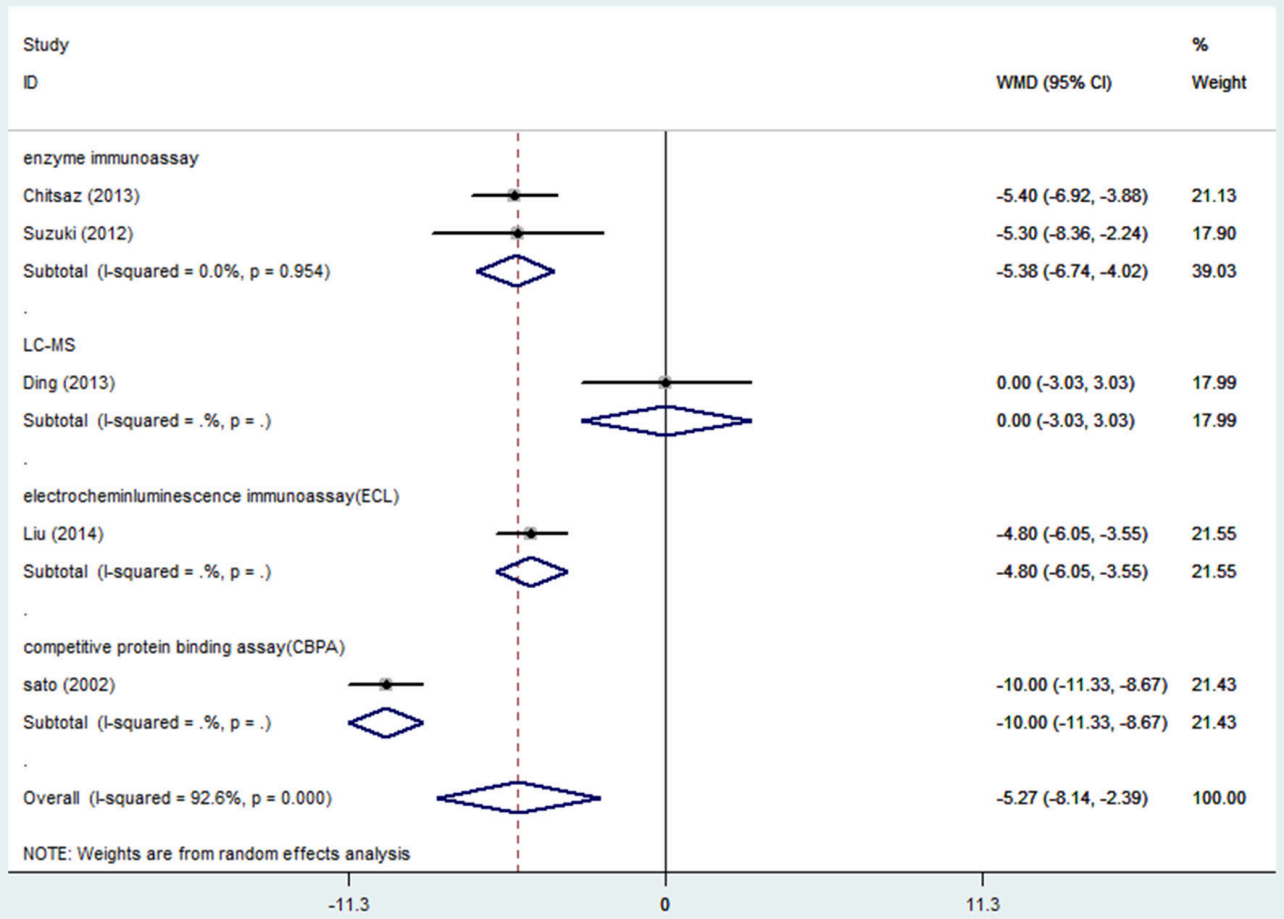
Forest plot of vitamin D levels in PD patients with different severity.

Six articles (16, 17, 19, 25, 26, 30) including 991 PD patients investigated the difference in serum vitamin D levels among different severity of PD groups based on H&Y scale. We only included the paper which provided more detailed descriptions (25) since the two articles (25, 26) were published by the same first author. Random effect model illustrated serum vitamin D concentrations were significantly low in the higher H&Y stages group (WMD-5.27, 95%CI −8.14, −2.39, Figure 6). The high degree of heterogeneity (*I*^2^ = 92.6%) could be greatly explained by assay methods. The publication bias and sensitivity analyses were not detected because of small number of studies.

### Correlative factors of serum vitamin D levels in PD

**Figure 7:**
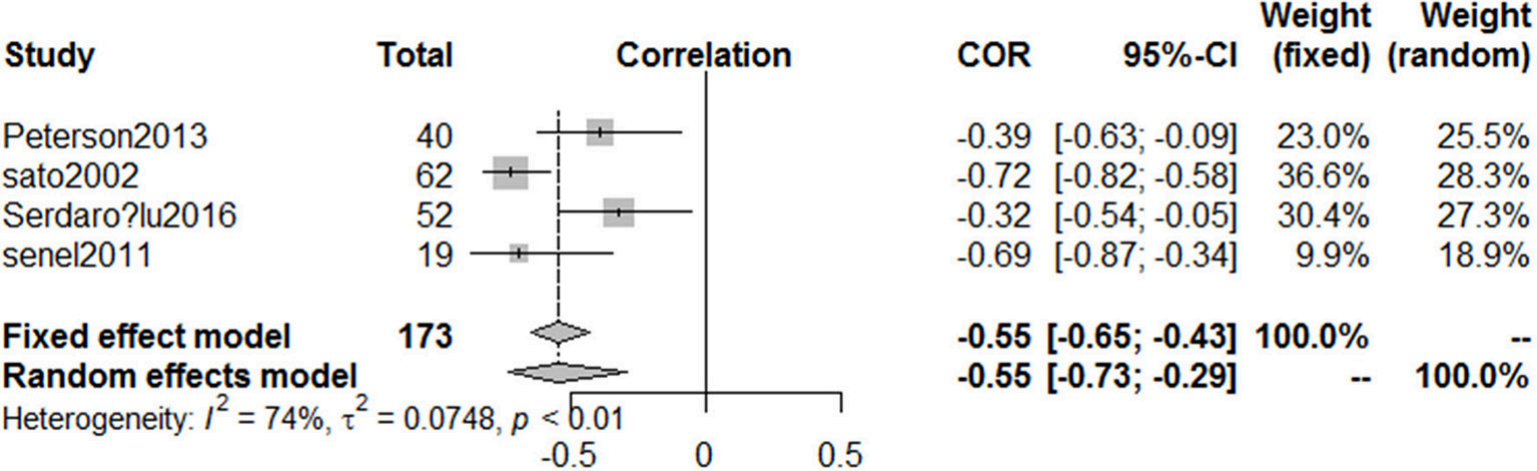
Forest plot of the correlation coefficient between vitamin D levels and different HYstages in PD patients.

**Figure 8:**
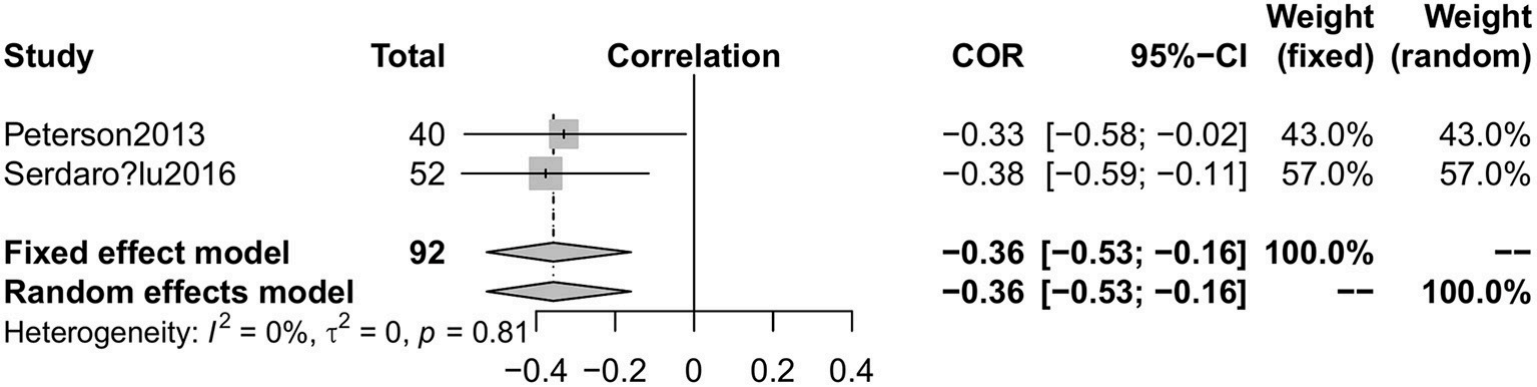
Forest plot of the correlation coefficient between vitamin D levels and mUPDRS scores in PD patients.

Five articles (22, 25–28) involving 244 PD patients explored the relationship between serum vitamin D concentrations and H&Y stages. Because the two articles (25, 26) were published by the same first author (Sato Y), we only included the more comprehensive one (25). The pooled correlation coefficient (spearman) was −0.55 but it displayed significant heterogeneity (95%CI −0.73, −0.29, *I*^2^ = 74%, Figure 7), so the random effect model was used. Furthermore, fix effect analysis of two studies (22, 28) which investigated the association of UPDRS III scores in PD patients with vitamin D levels indicated the pooled correlation coefficient (person) was −0.36 (95%CI −0.53, −0.16, *I*^2^ = 0%, Figure 8). However, serum vitamin D concentrations were not associated with duration of PD (19, 28) (*P* = 0.37) and age of patients (28) (*P* = 0.49). The sensitivity analyses showed consistent results and we could not make publication bias for finite studies.

## Discussion

Although three previous meta-analysis conducted the association between vitamin D status and PD (7–9), they did not analyze the relationship of vitamin D deficiency and the severity of PD. In addition, limited number of studies were included in the three previous meta-analysis, i.e., Lv et al. (7) recruited two studies (17, 18), Shen et al. (8) included three studies (17, 18, 33), and Rimmelzwaan et al. (9) included eight studies (15, 23, 26, 27, 31, 35–37). The current meta-analysis included large number of studies and not only assessed the association between serum vitamin D levels and the risk of PD but also the association between serum vitamin D levels and the severity of PD.

We found that the serum 25(OH)D levels in PD patients were significant lower than those in controls, which was consistent with the previous study (7). However, there was a high heterogeneity originating from assay methods. We further found that people who lived in higher latitude regions have lower serum 25(OH)D concentrates compared with people who lived in lower latitude regions, which is in accordance with the traditional theory that lower latitude area gets more sunlight which can enhance vitamin D biosynthesis. With regard to lower vitamin D levels in PD patients, we should admit limited mobility has an effect on low level of vitamin D due to less sunlight exposure in PD patients. However, high prevalence of deficient vitamin D concentrations also exist in non-disabling and early PD subjects even if they had normal ambulation (36). Wang et al. (33) reported serum vitamin D2 levels obtained mainly from diet rather than sunlight. Gastrointestinal dysfunction including dysphagia, delayed gastric emptying and constipation could result in poor digestion and absorption, which may also contribute to the low serum vitamin D levels in PD patients (38). In addition, malnutrition, failure of atrophic skin to produce vitamin D as well as comorbidities, such as renal or hepatic dysfunction in the elderly could also influence the vitamin D concentrations (39–41).

Both vitamin D insufficiency and deficiency can increase the risk of PD, however there is an increased risk of PD in vitamin D deficiency as compared to insufficiency vitamin D. The serum 25(OH)D levels were remarkably distinct among different severity PD groups. And they also were negatively correlated with H&Y stages and UPDRS III scores, but not the disease duration and the age of PD patients. Furthermore, the possible biological mechanisms for explaining vitamin D status contributing to the severity of PD are complicated.

First, it is of utmost importance to understand that only active vitamin D is responsible for all the normal physiological function inside our body. And the active process of vitamin D is regulated by 1α-hydroxylase in kidney, an enzyme which can change 25(OH)D into 1,25(OH)2D3. As for vitamin D receptor (VDR) mediated by special VDR genes, VDRs knockout mice showed significantly impaired motor functions (42). Both 1α-hydroxylase and VDR are expressed in human brain especially in the substantia nigra and hypothalamus (43). An animal study indicated that vitamin D increased tyrosine hydroxylase positive cells and inhibited inflammatory responses to ameliorate PD symptoms (44). Furthermore, single nucleotide polymorphisms (SNPs) in the VDR gene also have an effect on the serum vitamin D levels in PD patients (45, 46). Second, vitamin D plays an important role in neuroprotective actions through promoting the release of glial cell-derived neurotrophic factor (GDNF) and other trophic factors. Besides, vitamin D can indirectly restore dopaminergic circuits by increasing these neurotrophic factors. As a group of fat-soluble secosteroids, it is also easy to cross the blood brain barrier and exert its physiological effects (47). Third, lewy bodies are abnormal aggregates of alpha synuclein that develops in neurons in PD. Vitamin D analogs can decrease intracellular-free Ca (II) and downregulate the expression of calbindin-D28k to reduce α-synuclein aggregation (48). However, so far its pathogenesis has not been completely figured out.

There are some limitations in our meta-analysis that should be considered. Assay methods contributed largely to the discrepancy among these studies. There are many ways to detect the serum vitamin D levels which have different sensitivities and specificities (49). Till now there is no single ideal technique to synthesize the strengths of different test methods. Further attention should be paid in this area so that we can better explore the issue of vitamin D in PD. As for other confounding factors (e.g., sex, race, Body Mass Index, season, education, anti-PD drugs), we could not conclude anything else due to incomplete data. We only summarized the information from H&Y stages and UPDRS III scores, other indexes which were identically vital to assess PD symptoms, such as total UPDRS, UPDRS II, non-motor symptoms scale and so on. Relative studies are rare so that we could not do further analysis. We have to cautiously explain the finding of the relationship between vitamin D levels and duration of PD and age of PD patients. More studies are needed to verify our conclusion due to the small sample size in the meta-analysis. In addition, we did not analyze the association of PD with vitamin D from different sources i.e., vitamin D2 (dietary) and vitamin D3 (mainly sunlight). Finally, there are only a few studies which focused on the vitamin D supplement and its relation to PD (50–52), and only one double blinded RCT study (50). Our current meta-analysis cannot elucidate the role of vitamin replacement.

## Conclusion

In summary, our meta-analysis indicated that low serum vitamin D levels were directly correlated to the severity of symptoms of PD and increased risk of developing PD. Our results provide an incremental evidence to evaluate the role of vitamin D in the progression of PD. As a potential biomarker for PD, it should be considered to monitor the serum vitamin D levels. However, more high quality longitudinal randomized researches are urgently needed to address the necessary of vitamin D replacement in PD.

## Author contributions

XL, YT, and RO did the literature review. XL did the statistical analysis. All authors have significantly contributed discussions on the manuscript and reviewed the paper during the writing process.

### Conflict of interest statement

The authors declare that the research was conducted in the absence of any commercial or financial relationships that could be construed as a potential conflict of interest.
